# Editorial: Hunting for inflammation mediators: identifying novel biomarkers for autoimmune and autoinflammatory diseases, volume II

**DOI:** 10.3389/fimmu.2026.1994794

**Published:** 2026-09-22

**Authors:** Li Zeng, Jian Liu

**Affiliations:** 1 Shanxi Academy of Medical Sciences, Taiyuan, China; 2 School of Biochemistry and Biomedical Sciences, University of Bristol, Bristol, United Kingdom

**Keywords:** autoimmune, autoinflammation, biomarkers, inflammation, inflammation mediators

Building on the first collection on the topic, the second volume comprises seven original research articles, two case reports, one perspective, and one systematic review.

Three research papers focus on rheumatoid arthritis (RA)-related biomarkers and inflammatory mechanisms, spanning cell death, cardiovascular complications and undifferentiated arthritis (UA). PANoptosis, a coordinated convergence of apoptosis, pyroptosis and necroptosis, represents an inflammatory form of programmed cell death that may contribute to RA pathology. By integrating three GEO synovial-tissue datasets, Lu et al. identified 85 PANoptosis-related genes. Validation in RA fibroblast-like synoviocytes (RA-FLS) and an adjuvant-induced arthritis rat model highlighted IL-18, NLRP3, GBP1 and TNFSF10 as key differentially expressed genes (DEGs). While the study inferred a link between imbalanced synovial M0/M1 macrophage infiltration and PANoptosis-associated signature, another recent mechanistic study has demonstrated that protective macrophages themselves undergo bona fide PANoptosis and may constitute a principal cellular driver and amplifier of inflammation in RA synovial pathology (1).

Beyond joint pathology, the systemic inflammatory burden of RA can also affect the cardiovascular system. Jiang et al. found that collagen-induced arthritis (CIA) rats developed a sequence of cardiovascular changes associated with chronic systemic inflammation and inflammatory spillover from local joint inflammation, progressing from myocardial fibrosis to diastolic dysfunction and cardiac hypertrophy, and subsequently towards systolic dysfunction accompanied by lipid abnormalities and cardiac stress. These changes occurred before the apparent formation of atherosclerotic plaques in the aorta or coronary arteries, a conventional indicator of atherosclerotic disease, highlighting the importance of early detection, monitoring and intervention for inflammatory cardiomyopathy (2).

At an earlier stage of disease, identifying biomarkers that can distinguish patients with UA who will evolve into a defined rheumatic disease remains a clinical challenge. Alongside ACPA, rheumatoid factor (RF), acute-phase reactants and imaging findings, novel diagnostic and prognostic biomarkers could improve disease classification and monitoring during this heterogeneous phase of joint inflammation. Higher serum and synovial fluid levels of Galectin-1 (Gal-1) were previously documented in patients with early RA or UA in the PEARL cohorts, although it did not distinguish RA from UA (3). In a two-year prospective observational study, Valero-Martinez et al. further reported that serum Gal-1 did not predict disease progression or activity in 139 patients with UA. Baseline Gal-1 levels were not associated with eventual diagnosis, disease severity, antibody status, disability, or treatment intensity, and remained largely stable over time despite improvements in disease activity. Thus, Gal-1 may be associated with the presence of early inflammatory arthritis, but does not appear to be specific for RA or predictive of subsequent clinical trajectory of UA.

The collection extends this biomarker perspective beyond rheumatic disease. In Aicardi–Goutières syndrome (AGS), a prototype I interferon (IFN)-driven neuroinflammatory disorder, conventional IFN scores may not adequately reflect the extent of neurological injury. Wege et al. identified plasma neurofilament light chain (pNfL) and glial fibrillary acidic protein (pGFAP) as sensitive markers of neuroaxonal and astroglial injury, respectively. Both markers correlated with disease severity and declined following JAK inhibitor treatment, whereas the IFN score remained unchanged, suggesting that they may provide more direct and quantitative measures of neurological disease burden and treatment response, particularly in children in whom neurological assessment can be challenging.

Biomarker-guided diagnosis is also important when conventional serological testing may be insufficient. Tarancon-Diez et al. studied 86 patients with unexplained iron deficiency (ID) and found a high prevalence of previously underrecognized coeliac disease (CD), with 14% having confirmed CD and a further 39.5% meeting the authors’ criteria for suspected seronegative CD with Marsh 1 lesions. These findings reinforce the importance of investigating CD in patients with unexplained ID, even in the absence of anaemia, while recognising that seronegativity does not completely exclude CD when clinical suspicion remains high (4).

IgG4-related ophthalmic disease (IgG4-ROD) is increasingly recognised as a complex immune-mediated fibro-inflammatory disorder. Using high-throughput 4D-DIA proteomics of lacrimal gland biopsies from patients with confirmed or suspected IgG4-ROD and controls, Ma et al. characterised molecular alterations associated with fibro-inflammatory pathology, extending current understanding beyond IgG4-positive plasma-cell infiltration by implicating elevated innate immune sensing through TLR8/NF-κB signaling and identifying MMP7, periostin (POSTN) and CD163 as potential contributors to fibrotic remodelling. These findings are consistent with a previous animal study of IgG4-ROD suggesting CD163^+^ M2 macrophage and TLR7/IRAK4/NF-κB signalling in the induction of profibrotic cytokines (5), although a different Toll-like receptor was implicated.

In the booming field of mesenchymal stromal cell (MSC) therapy, mechanism-associated biomarkers may help bridge the gap between biologically heterogeneous MSC products and reproducible therapeutic outcomes (4). Using multi-omics and single-cell analyses, Yang et al. associated modulation of *S100A9*, *MMP9* and *BACH2* with the therapeutic effects of human amniotic MSCs (hAMSC) in an imiquimod-induced mouse model of psoriasis. These changes accompanied reduced skin inflammation and epidermal hyperproliferation and may involve suppression of systemic IL-17/TNF-α signalling and keratinocyte ROS production.

The two case reports in this collection share a common theme: biomarkers that strengthen diagnostic confidence and guide treatment selection. Zhou and Guo proposed intrathecal IgG synthesis as an additional layer of information in anti-GQ1b antibody syndromes, rather than relying on CSF protein elevation or albuminocytologic dissociation alone. Similarly, Liedtke et al. highlighted the importance of confirming rare antisynthetase antibodies (e.g. anti-Ha) by protein immunoprecipitation to improve diagnostic confidence and overcome uncertainty associated with commercial multiplex assays in rapidly progressive interstitial lung disease (ILD).

From a clinical perspective, Cheng et al. examined the diagnostic distinction between idiopathic multicentric Castleman disease (iMCD) and systemic lupus erythematosus (SLE), two conditions with overlapping systemic inflammatory manifestations and laboratory abnormalities. Persistent constitutional symptoms, lymphadenopathy and atypical serological findings should prompt consideration of Castleman disease and other autoimmune mimickers, particularly when the clinical course is inconsistent with SLE or the response to immunosuppression is suboptimal.

Finally, a systematic review and meta-analysis by Guan et al. quantitatively investigated the association between antiphospholipid antibody (aPL) profiles and renal injury in SLE, searching the literature up to September 12. 2025. Across 70 studies involving 12,456 patients, aPL-positive SLE patients had approximately two-fold higher odds of renal injury, with the strongest association observed for lupus anticoagulant and anticardiolipin antibodies. In the context of contemporary SLE management, in which renal involvement is a major prognostic determinant (6), further prospective studies are needed to determine the merit of aPL status for renal risk stratification, particularly in view of the heterogeneity and observational nature of the available evidence.

Autoimmune and autoinflammatory diseases are remarkably diverse, making them challenging to diagnose, monitor and treat. Together with the first volume, this collection highlights the growing promise of biomarkers to illuminate disease mechanism, refine diagnosis and prognosis, monitor disease activity, and guide therapeutic responses.

The quest goes on.
